# Investigating the relationship between resilience and professional ethics in nurses: a cross-sectional study in southern Iran

**DOI:** 10.1186/s12912-023-01578-1

**Published:** 2023-10-30

**Authors:** Yasaman Asadi, Zahra Molazem, Zinat Mohebbi, Parvin Ghaemmaghami

**Affiliations:** 1 Student Research Committee, Shiraz University of Medical Sciences, Shiraz, Iran; 2 Department of Nursing, School of Nursing and Midwifery, Shiraz University of Medical Sciences, Shiraz, Iran

**Keywords:** Resilience, Professional ethics, Nursing

## Abstract

**Introduction:**

All businesses that deal directly with clients need to observe ethics, but in the nursing profession due to its nature, compliance with professional ethics becomes more necessary. On the other hand, nurses face tensions and difficult conditions in their daily work environment. Resilience is one of the most important abilities of human beings, which in difficult conditions causes effective adaptation to stress-causing factors. Therefore, the relationship between resilience and professional ethics becomes important.

**The aim of the study:**

The present study was conducted to investigate the relationship between resilience and professional ethics in nurses of Shahid Ganji Hospital, Borazjan 2022–2023.

**Methods:**

The current study is a cross-sectional descriptive-analytical study. The study environment was Shahid Ganji Hospital in Borazjan, Bushehr, Iran. The research method of the current study was census, so all 400 nurses of this hospital were invited to participate in the study. Finally, 340 of them were included in the study based on the inclusion criteria. Data collection tools included a demographic information form, Connor-Davidson questionnaire, and Cadozier professional ethics questionnaire. Data analysis was done using SPSS version 22 software.

**Results:**

Most of the participants were between 25 and 30 years old. The average overall score of resilience and professional ethics of nurses participating in the study was 64.1 ± 16.3 and 34.3 ± 9.4, respectively. No significant difference was observed between the demographic characteristics of nurses with resilience and professional ethics. The Spearman correlation coefficient indicated a statistically significant and inverse relationship between professional ethics and resilience levels (r = -0.265, P < 0.001).

**Conclusion:**

The present study demonstrated a significant and inverse relationship between professional ethics and resilience among nurses. Furthermore, professional ethics were at a moderate level, while resilience was at a good level. Therefore, it is recommended that enhancing professional ethics skills through the organization of educational workshops for nurses should be considered by nursing managers.

## Introduction

The discussion of ethics is one of the concepts that has received a lot of attention these days. For many professions, various principles, rules, and ethical standards have been developed at the organizational, national, and even global levels under the title of professional ethics, which the owners of that profession are required to comply with. Professional ethics is considered a basic element in all health professions, including nursing, and it plays an important role in the moral behavior of nurses with patients and strongly affects the improvement of patients’ health [1].

Nursing ethics is the observance of professional ethics in providing nursing care. Therefore, compliance with ethics in nursing technical functions is more sensitive and important than general issues of care [2]. Professional ethics is a type of moral commitment and work conscience towards any kind of work, duty, and responsibility; thus, ethics is recognized as an important part of providing care [3]. People’s ethics cause positive or negative consequences at the level of organizations. In the discussion of professional ethics, one of the important elements in the process of development, productivity, and improvement of the organization’s performance is having a high work commitment. Professional ethics is one of the basic issues of all human societies. In the West, in the knowledge related to management and organization, there is a branch called professional ethics [4]. Professional ethics has been raised as a basic and important issue in clinical sciences and medical education, and in the last two decades, discussion and research on it has been at the forefront of medical education research [5]. Nurses are the largest service provider group in the medical system and have a great impact on the quality of healthcare and compliance with professional ethics standards in order to provide quality care [6]. In general, people working in the health field always face many stressors. Studies show that the level of this tension in the nursing profession is higher than other health professions [7, 8]. Nurses experience stress due to special job situations and facing many unfortunate cases in the work environment, which is dangerous and can cause damage to their health [9]. The stressful nature of the nursing profession poses a serious threat to the mental health of nurses and in the long run, it can cause their job burnout [10].

Several studies have been conducted regarding the degree of observance of the principles of professional ethics from the point of view of nurses, and professional ethics have been examined in different departments [4, 11]. In these studies, the highest score was related to professional ethics in providing services, and in general, the observance of ethical principles by nurses was average [1]. Studies showing the views of patients towards nursing services had different results, while some showed the appropriate quality of nurses’ treatment [12], others showed that the nursing services provided could not meet the demands and expectations of patients [6]. In a report, it was found that moral conditions and atmosphere can increase effective resilience and subsequently lead to an increase in nurses’ performance [13].

Resilience is defined as social and individual ability to recover, adapt and sustain in response to adverse conditions [14]. According to Lachman, resilience helps people to identify moral dilemmas and express them effectively and take necessary actions [15]. People with a high level of resilience are more likely to experience positive emotions in their lives and have higher self-confidence [16]. By improving resilience, a person can resist and overcome stressful and anxiety-inducing factors, as well as factors that cause many psychological problems [17, 18]. Resilience is very important for nurses to reduce negative outcomes and increase positive outcomes of stress [19].

American Association of Colleges of Nursing (AACN) consider the resilience capacity necessary for the nursing job position, because in their opinion, otherwise the working conditions will be very difficult and will lead to psychological and biological injuries for nurses and the quality of care [20]. Research has shown that resilience as an important factor improves nurses’ satisfaction and job longevity in different departments [21–23].

Resilience in nurses is achieved through knowledge, skill, and clinical experiences, which leads to the self-confidence and flexibility of nurses in dealing with and adapting to complex work environments [24]. Enhancing resilience may also help nurses reduce moral conflict [25]. In a study conducted by Salimi et al. (2017), the resilience of nurses was reported at a relatively high level and equal to 63.24 ± 14.80 [17].

Talebian et al. (2022) found that when moral distress increases, nurses use resilience mechanisms [26]. Abdollahi et al. (2021) also reported that creating a suitable moral climate can increase resilience in nurses [13]. This is while Graminejad et al. (2020) found that there is no significant relationship between the desire to leave the profession and the level of resilience [27]. Therefore, considering the importance of resilience and professional ethics in nurses, the present study was conducted to determine the relationship between resilience and professional ethics among nurses at Shahid Ganji Hospital in Borazjan 2023.

## Method

### Design, settings, and participants

The current study is a cross-sectional descriptive-analytical study. The research population was all nurses working in Shahid Ganji Hospital of Borazjan under the supervision of Bushehr University of Medical Sciences, Bushehr, Iran. This research was conducted for 3 months from December to February 2022–2023.

The research environment of the present study was Shahid Ganji Hospital in Borazjan. This healthcare facility serves as the establishment for providing health care and emergency medical services for patient safety within the boundaries of Dashtestan City. To achieve a better result, all available nurses in the hospital were included in the study. The inclusion criteria were willingness to participate in the study, having at least one year of work experience, and having a bachelor’s or master’s degree in nursing. The exclusion criteria were the participant suffering from psychological problems and being treated by a psychiatrist according to the individual’s self-report, unwillingness to participate in the study, and incomplete questionnaires.

### Data collection tools

#### Demographic information

Demographic information included age, gender, marital status, employment status, work experience, level of education, rotating or fixed shift.

#### Connor-Davidson questionnaire

This tool was designed by Connor and Davidson (2003) [28]. It contains 25 items that are scored on a Likert scale (completely false = 0, rarely true = 1, sometimes true = 2, often true = 3, always true = 4). Therefore, the range of test scores is between 0 and 100. Higher scores indicate greater resilience of the subject. The cut-off point for the total score of the questionnaire is 50 [29]. This test has 5 factors: perception of individual competence, trust in individual instincts, tolerance of negative emotions, positive acceptance of change and secure relationships, control and spiritual influences. The psychometric properties of this scale have been investigated in six groups, the general population, primary care patients, psychiatric outpatients, patients with generalized anxiety disorder, and two groups of post-traumatic stress patients. The producers of this scale believe that this questionnaire is well able to distinguish resilient from non-resilient people in clinical and non-clinical groups and can be used in research and clinical situations. Also, the reliability coefficient obtained from the retest method in a 4-week interval was 0.87 [30]. This scale has been standardized in Iran by Zarifi et al. (2016). He used Cronbach’s alpha method to determine the reliability of this tool and reported a reliability coefficient of 0.77 [31]. In the research conducted by Samani et al. (2007) among the students, they reported its reliability as 0.93, and the validity (using the method of factor analysis and convergent and divergent validity) was verified by the creators of the test in different normal and at-risk groups [30].

#### Professional ethics questionnaire

The professional ethics questionnaire was designed by Cadozier (2003) [32]. It has 16 items and 8 components (responsibility, honesty, justice and fairness, loyalty, superiority and competitiveness, respect for others, sympathy with others, and respect for social values and norms). The questions are scored on a Likert scale (1 = very little to 5 = very much). A score between 16 and 32 indicates weak professional ethics, a score between 32 and 48 average professional ethics, and a score above 48 strong professional ethics. The validity and reliability of this questionnaire were confirmed in Iran by Jafari and Hanifi (2019) and Cronbach’s alpha was 0.88 [33].

### Ethical considerations

This study was reviewed and approved by the Ethics Committee of Shiraz University of Medical Sciences and has the ethical code “IR.SUMS.NUMIMG.REC.1401.088”. It was explained to the participants that participation in the research was optional and due to the confidentiality of the information, their names would not be included in the questionnaire. In addition, informed consent was obtained from the participants according to the Helsinki Convention.

### Data analysis

In this research, mean (and standard deviation) was used to describe quantitative data, and frequency (percentage) was used to describe qualitative data. Descriptive statistical methods (prevalence, percentage, mean, and standard deviation) were used to describe the data, and independent t-test, one-way variance, Tukey’s post hoc test, and Pearson’s correlation coefficient were used to determine the relationship between variables and differences based on demographic characteristics. Meanwhile, in this study, based on the central limit theorem, parametric tests were used to examine all the objectives. The analysis of the study was done by SPSS version 22 software.

## Results

### Demographic characteristics

Of the 400 nurses who were invited to participate in the study, 40 did not agree to participate and 20 questionnaires were excluded from the study due to not being completed, and finally, the questionnaires of 340 nurses were included in the study (response rate = 85%). The demographic characteristics of nurses are shown in Table 1.

**Table 1:** Demographic characteristics of nurses participating in the study (n = 340)

Variable		Frequency	Percent
Gender	Female	221	65.0
	Man	119	35.0
Age	25–30	155	45.6
	30–35	90	26.5
	35–40	62	18.2
	> 40	33	9.7
Marital status	Single	143	42.1
	Married	197	57.9
Level of education	Bachelor	305	89.7
	Master of Science	35	10.0
Shift work	Fixed	37	10.9
	Rotating	303	89.1
Work Experience	< 5	155	45.6
	5–10	90	26.5
	10–15	62	18.2
	> 15	33	7.9
Employment Status	Official employment	190	55.9
	Contractual employment	34	10.0
	Part time	35	10.3
	Short time employment	81	23.8

The findings showed that the average overall score of professional ethics of the nurses participating in the study was 34.3 ± 9.4 with a range of 16 to 77, and the average score of resilience of the nurses participating in the study was 64 ± 1.16 with a range of 10 to 100.

According to the t-test (independent), no significant relationship was observed between the variables of gender, marital status, shift work, and education level with the resilience score. Also, the analysis of the variance test showed that there is no statistically significant relationship between the age group, work experience, and employment status of nurses with the resilience score. (Table 2)

**Table 2:** Comparison of resilience scores of nurses participating in the study based on demographic characteristics (n = 340)

Variable			Mean	Standard deviation	P
Gender	Female		64.4	15.8	0.522*
	Man		63.3	17.2	
Age		25–30	64.0	16.4	0.081**
		30–35	62.8	16.7	
		35–40	70.7	17.3	
		> 40	64.1	16.3	
Marital status		Single	63.9	16.3	0.926*
		Married	64.1	16.3	
Education		Bachelor	63.9	16.3	0.603*
		Master of Science	65.4	16.4	
Shift work		Fixed	63.3	20.4	0.777*
		Rotating	64.1	15.8	
Work Experience		< 5	64.0	16.4	0.081**
		5–10	62.8	16.7	
		10–15	62.4	14.3	
		> 15	70.7	17.3	
Employment Status		Official employment	63.4	16.8	0.136**
		Probationary employment	56.8	15.3	
		Contractual employment	61.1	16.5	
		Part time	65.8	16.0	
		Short time employment	64.0	16.3	

*: t-test, **: analysis of variance (ANOVA)

There was no statistically significant correlation between the resilience score and the demographic characteristics of the nurses. According to the t-test (independent), no significant relationship was observed between the variables of gender, marital status, shift work and education level with the professional ethics score. Also, the analysis of variance test showed that the age group, work history, and employment status of the nurses had no statistically significant relationship with the professional ethics score. (Table 3)

**Table 3:** Comparison of professional ethics scores of nurses participating in the study based on demographic characteristics (n = 340)

Variable			Mean	Standard deviation	P
Gender	Female		34.4	8.6	0.913*
	Man		34.4	10.8	
Age		25–30	34.2	9.1	0.080**
		30–35	36.2	10.0	
		35–40	33.3	10.3	
		> 40	31.7	6.4	
Marital status		Single	34.8	9.6	0.442*
		Married	34.0	9.4	
Education		Bachelor	34.4	9.4	0.637*
		Master of Science	33.6	10.0	
Shift work		Fixed	32.2	8.3	0.147*
		Rotating	34.6	9.5	
Work Experience		< 5	34.2	9.1	0.080**
		5–10	36.2	10.0	
		10–15	33.3	10.3	
		> 15	34.3	9.4	
Employment Status		Official employment	34.5	9.8	0.210**
		Probationary employment	40.1	10.9	
		Contractual employment	34.1	8.4	
		Part time	33.3	8.7	
		Short time employment	34.3	9.4	

*: t-test, **: analysis of variance (ANOVA)

### Determining the relationship between the average score of professional ethics and resilience in nurses

Considering that according to Kolmogorov-Smirnov Test, the score of professional ethics and resilience did not follow a normal distribution (P < 0.001), Spearman correlation coefficient indicated a statistically significant and inverse relationship between professional ethics and resilience levels (r = -0.265, P < 0.001). In other words, as the score of professional ethics increases, the level of resilience decreases and vice versa.

## Discussion

The present study has been conducted with the general aim of determining the relationship between resilience and professional ethics in the nurses of Shahid Ganji Hospital in Borazjan, South of Iran in 2022–2023. The findings of the study showed that the resilience score of nurses was at a good level, while the overall score of professional ethics was average. Also, the results showed that there was an inverse and significant relationship between professional ethics and resilience in nurses.

The present study showed that the average overall resilience score of the participating nurses was a good level and the resilience score did not show a significant relationship with any of the demographic variables.

Guo et al. (2018) found that the level of resilience of nurses was at an average level [34]. Hegney et al. (2015) reported that the mean resilience score for Australian nurses was in the average level, which was somewhat lower than that of nurse leaders and other community samples [35].

Manomenidis et al. (2019) reported that resilience among nurses was at an average level [36]. Also, they showed that education helps to increase nurses’ resilience. A higher level of education indicates higher academic independence and characteristics that may help nurses cope with workplace adversities [37].

In the present study, the average resilience of the participants with a master’s degree is higher than that of a bachelor’s degree. But this difference is not significant. The results are in line with the study of Manomenidis et al. (2019), yet in terms of meaning, it is not aligned. Perhaps the reason for this difference is the sample size of the aforementioned study which is 3 times more than that of the present study. And when the sample size is higher, the differences between different groups are more visible.

Aqtam et al. (2023) reported that the level of resilience of nurses was low and the resilience of female nurses was lower than that of men [38]. Afshari et al. (2021) also found that women had significantly less resilience than men [39]. This is while in the present study, women showed more resilience. Therefore, these results are not consistent with the results of the present study. This difference can be attributed to the sampling time. Unlike the current study, the two mentioned studies were conducted during the peak of the Covid-19 crisis, and increased worry in women has caused a noticeable decrease in resilience compared to men.

The present study showed that resilience in married nurses is slightly higher than that of single nurses. But this difference is not statistically significant. The results of Afshari et al.‘s (2021) study were similar to the present study [39]. They also found that resilience in married nurses was more than in unmarried nurses and it was not significant as in the present study.

A study on the Covid-19 pandemic conducted by Saddik et al. (2021) also showed that the level of anxiety of healthcare workers was relatively high and the most concern was related to the infection of family as well as the health consequences of this disease [40]. In fact, the lack of psychological and social support and the feeling of inability to fulfill their responsibility towards children and family probably play an important role in reducing nurses’ resilience during the Covid-19 pandemic [41]. Therefore, it can be one of the reasons for the low level of resilience in single nurses in the present study.

The present study showed that resilience increases with age, education, and work experience. But in all three cases, these differences are not significant. The findings can be explained by the fact that as the age, work experience, and education of nurses increase, they experience more exposure to stressful situations and develop their abilities and skills to deal with critical situations. The development of such skills helps to create different coping strategies, which can facilitate their adaptation and enable them to act more effectively and flexibly in such situations [42, 43]. Afshari et al. (2021) conducted a study on the resilience of operating room nurses and reported that as the nurses’ age, experience, and education increase, their resilience improves [39].

Another study conducted in Iran on the mental health and job satisfaction of nurses in the face of Covid-19 showed that older medical staff had better mental health and the level of education was a predictor of physical and mental health [44]. Although the present study was not conducted during the peak of the Covid-19 epidemic, other studies show that the increasing age and experience of nurses leads to an increase in their skills in managing stress at work [45, 46]. Therefore, to increase the resilience of medical staff with lower education and work experience, it is very necessary to provide them with relevant training that can increase their knowledge and skills in managing and coping with disasters like covid-19.

The present study showed that the average score of the professional ethics of the nurses participating in the study was average, and the professional ethics score did not show a significant relationship with any of the demographic variables.

In explaining this finding, it can be pointed out that the level of professional ethics in the researched nurses is influenced by many factors including the cultural atmosphere and the research area, the nurses’ motivation to continue working, the type of employment, the level of attention of the organization to the demands of the nursing community, and their mental and emotional state. These factors can affect the nurses’ commitments to the organization.

Sadrollahi and Khalili (2014) in a study titled investigating the correlation of organizational commitment with the sensitivity of professional ethics in nurses in the west of Golestan Province, concluded that the level of sensitivity of professional ethics in nurses was average [47]. Perhaps the reason for the average level of professional ethics in nurses can be related to their awareness of compliance with ethical standards. Because, as it was shown in the study by Roshanzadeh et al. (2022), the efforts of systems to improve nurses’ awareness can play an effective role in improving their performance in line with ethical standards in the clinical environment [48]. Kalvandi et al. (2019) also reported the observance of ethical principles in nurses at an average level in their study [1]. In the research by Khaki et al. (2015) 48.3% of patients evaluated nurses’ performance at an average level of compliance with professional ethics [49]. Maarefi et al.‘s research (2013) also showed that the total average score for morality was 20.68 out of 28, which was at the average level [50]. The results of these studies are in line with the results of the present study. Nursing managers need to make effective and useful plans to improve the professional ethics of nurses.

The present study showed that there was a significant inverse relationship between professional ethics and resilience. In other words, as the level of professional ethics increased, the level of resilience decreased, and vice versa. These findings align with Sala Defilippis et al.‘s (2019) assertion that ethics and resilience can often complement each other and both are crucial for the effectiveness and overall well-being of nurses [51]. Rushton et al. (2021) reported a positive correlation between resilience and ethical competence. In other words, increasing resilience led to an increase in ethical competence among nurses [52]. On the other hand, Clark et al. (2021) found in their study that the level of resilience did not have a significant relationship with ethical distress [53]. Additionally, Arries-Kleyenstuber (2021) also reported that there was no significant association between resilience and ethical ideology [54].

Recent study results do not align with the findings of the present study. It might be possible to elucidate the findings of the current study by considering that the level of professional ethics is average, and the level of resilience is good. It seems that inappropriate education could be a reason for the inverse relationship between these two variables. The methods used to enhance resilience may not be in harmony with ethical principles, or they may unintentionally promote behaviors that do not align with ethical standards. For example, if resilience training solely focuses on personal coping mechanisms without considering ethical decision-making, it might lead to a decrease in professional ethics. Furthermore, if the methods used to increase resilience primarily focus on nurses’ encounters with high-stress situations, it might inadvertently lead to desensitization or occupational burnout, potentially reducing sensitivity to ethical concerns. Additionally, nurses’ individual characteristics, such as values and personal experiences, may play a role in these findings.

On the other hand, nurses who prioritize professional ethics may invest more time and energy in addressing ethical concerns, advocating for patient rights, and adhering to ethical guidelines. This increased focus on ethical considerations potentially leaves them with less time and energy to cope with personal stressors, which may reduce their flexibility. Moreover, ethical dilemmas and moral distress can have a significant psychological impact and potentially lead to symptoms of occupational burnout, fatigue, or stress, all of which can reduce resilience.

Another possible reason for the lack of a direct relationship between professional ethics and resilience could be that as nurses gain more experience and expertise, they may become less indifferent to adhering to ethical principles, and conversely, less experienced individuals may, due to being more influenced by stressors, try to adhere more to ethical principles.

Chenari and Behzadi (2019) also considered professional ethics as influential in enhancing some components of resilience. These findings do not align with the results of the current study. However, it should be noted that Chenari and Behzadi’s (2019) research was based on the experiences of researchers and books. In contrast, the present study was descriptive-analytical in nature and employed a questionnaire-based sampling method among nurses [55].

### Research limitations and suggestions for future research

Considering that the present study was conducted in a teaching hospital, the generalizability of the results to private hospitals should be approached cautiously. Furthermore, the use of self-report instruments could be one of the other limitations of this study. Also, it is suggested to conduct longitudinal studies to investigate how resilience and professional ethics change over time, using random samples. Conducting qualitative studies to investigate deeper feelings, experiences and the context of factors affecting resilience and professional ethics and conducting similar studies in other regions of the country are suggested to generalize the results to larger societies due to the different geographical regions, socio-economic status and cultures.

## Conclusion

The present study revealed that the professional ethics of nurses were at a moderate level, while resilience among nurses was at a good level. Additionally, professional ethics and resilience exhibited a significant and inverse relationship. The findings of this study can enhance our knowledge of the occupational resilience and professional ethics of nurses at a specific level. Furthermore, ongoing educational programs can play a crucial role in sensitizing nurses to ethical issues, thus improving their performance in line with ethical standards and enhancing their professional ethics. To better understand the results of this study, further research, including qualitative studies to explore the experiences and perceptions of nurses, is essential. Additionally, a cautious interpretation and generalization of research findings and potential limitations are of great importance.

## Data Availability

The datasets used and/or analyzed during the current study are available from the corresponding author on request.
